# Increase in condom use and decline in prevalence of sexually transmitted infections among high-risk men who have sex with men and transgender persons in Maharashtra, India: Avahan, the India AIDS Initiative

**DOI:** 10.1186/1471-2458-14-784

**Published:** 2014-08-03

**Authors:** Shreena Ramanathan, Sucheta Deshpande, Abhishek Gautam, Dilip B Pardeshi, Lakshmi Ramakrishnan, Prabuddhagopal Goswami, Rajatashuvra Adhikary, Bitra George, Ramesh S Paranjape, Mandar M Mainkar

**Affiliations:** FHI 360 India, H-5 Green Park Extension, New Delhi, 110016 India; National AIDS Research Institute (ICMR), Pune, Maharashtra India; Formerly with FHI 360, New Delhi, India; FHI 360 Headquarters, Washington, USA

**Keywords:** MSM, Transgender, Maharashtra, Avahan, Condom use, STIs, HIV

## Abstract

**Background:**

The present study assessed coverage, changes in condom use, and prevalence of HIV and other STIs among high-risk men who have sex with men (HR-MSM; highly visible, recruited from cruising sites/sex venues) and transgender (TG; male-to-female transgender persons, also called hijras) in the Indian state of Maharashtra.

**Methods:**

Data from Avahan’s computerized management information system; two rounds of integrated behavioral and biological assessment (IBBA) surveys (Round 1 with 653 HR-MSM/TG and Round 2 with 652 HR-MSM/TG); and project-supported condom social marketing was used for the present analysis. Logistic regression models were used to assess changes in key indicators over these two rounds and to explore the association between exposure to Avahan interventions and condom use and STI prevalence in HR-MSM/TG.

**Results:**

By December 2007, Avahan had reached about 90% of the estimated HR-MSM/TG population, and 83% of the estimated total population had visited STI clinics by March 2009. Free direct condom distribution by Avahan program NGOs and social marketing outlets in Maharashtra increased from about 2.7 million condoms in 2004 to 15.4 million in 2008. HR-MSM/TG were more likely to report higher consistent condom use (adjusted odds ratio [AOR]: 1.90; 95% confidence interval [CI] 1.01-3.58) with regular male partners (spouse/lover/boyfriend) in Round 2 of IBBA, compared to Round 1. HR-MSM/TG exposed to Avahan interventions were more likely to report consistent condom use with regular male partners (AOR: 2.46; CI 1.34-4.52) than those who were unexposed. Prevalence of reactive syphilis serology declined significantly from 8.8% in Round 1 to 1.1% in Round 2 (p = 0.001), while the observed change HIV prevalence (12.3% to 6.3%, p = 0.16) was insignificant.

**Conclusion:**

The current evaluation provides evidence for successful scale up and coverage of target population by Avahan interventions in Maharashtra. The assessment findings showed improved accessibility to condoms and reduced risk behaviours with male sexual partners. Syphilis prevalence declined; however HIV prevalence did not change and is still a major concern. Continued strengthening of core programmatic strategies are needed to effectively improve condom use with all partner types and to help bring sustained reductions in HIV risk in HR-MSM/TG and its onward transmission.

## Background

An estimated 2.47 million people were living with HIV in India in 2008 [1]. HIV epidemic in India has been concentrated among female sex workers (FSWs); men who have sex with men (MSM), including transgender (TG) persons; and injecting drug users (IDUs) [2]. Though the epidemic in India is largely due to heterosexual transmission, studies across the country have shown that sexual activity between men is relatively common [3–6] and HIV among MSM/TG populations is increasingly becoming an important route of transmission [2]. In India male-to-male sexual behavior takes place in diverse contexts, and MSM are known to have multiple partners, use condoms selectively during anal sex with male and female partners, and have high consumption of alcohol and drugs [4, 5, 7–12]. Social stigma and clandestine behavior add to the vulnerability of MSM/TG, making them an important target group for HIV prevention interventions. Estimated HIV prevalence in MSM/TG in India stood at 4.4% in 2010–2011, down from 7.4% in 2007. However, HIV prevalence in Maharashtra (9.91%) is much higher than the national average, and in 2007, Maharashtra (11.8%) had the second largest burden of the HIV infected in India [13, 14]. Most studies have consistently found HIV prevalence in MSM/TG to be higher than 5% in Maharashtra. Notably, studies have shown that MSM/TG in the country’s metropolitan cities, such as Mumbai, Chennai, Pune, and Bangalore show higher HIV prevalence [4, 10, 11, 15, 16].

The country’s National AIDS Control Organisation (NACO) has, since 1992, implemented targeted interventions for high-risk groups such as FSWs and men attending STI clinics. Maharashtra was among the first states in India to initiate targeted interventions for MSM [17]. Avahan: the India AIDS Initiative, funded by the Bill & Melinda Gates Foundation, was launched in 2003 with the aim of reducing the spread of HIV; it has since played a major role in supporting prevention interventions in the country [18]. Avahan worked in 83 districts of the country’s six high-HIV prevalence states among high-risk groups. One of these six states was the western state of Maharashtra, where Avahan covered 15 districts out of the total 35 districts; in eight of these districts Avahan was the sole provider of interventions (Table 1). Avahan’s strategies were designed to achieve high coverage (80% of the total estimated target population) through delivery of a package of proven prevention services that would address proximal and distal determinants of HIV risk [19, 20]. The key elements of the combination prevention program for HR-MSM/TG included peer-led outreach to promote behavior change, clinical services to treat STIs, provision of commodities (condoms and lubricants) for safe sex, support for community mobilization, and advocacy to reduce structural barriers to safer sexual practices [17]. Avahan’s intervention focus was on highly visible HR-MSM/TG in high-risk locations (cruising sites/sex venues, such as parks, local train stations, and bus stops), who tend to have a large number of sexual partners and regularly practice receptive anal sex or sell sex. They are therefore referred to as high-risk MSM [17]. The present analysis, using multiple data sources, was conducted to assess the overall effect of the Avahan program on HIV prevention in Maharashtra’s HR-MSM/TG population. The specific objectives of this paper are to examine the scale-up and coverage of the Avahan program, changes in condom use patterns and prevalence of HIV and other STIs, and the association between these outcomes and exposure to Avahan program among HR-MSM/TG in Maharashtra.

**Table 1:** **District-level intended Avahan coverage and intervention in Maharashtra for high-risk men who have sex with men and transgendered persons (HR-MSM/TG)**

Districts	District population^1^(thousands)	Estimated size of HR-MSM/TG in districts^2^	Avahan intended coverage^3^	History/type of intervention coverage*	IBBA sample^a^	
					Round 1	Round 2
Ahmednagar	4,088	534	100%	First and solo		
Beed	2,161	791	84%	First and solo		
Jalgaon	3,679	594	100%	First and solo		
Kolhapur	3,515	596	100%	First and solo		
Latur	2,080	256	48%	First and solo		
Mumbai	13,830	14,266	21%	Not first and minor	400^+^	373^+^
Thane	8,131	800	12%	No Avahan intervention		
Nandurbar	1,309	41	100%	First and solo		
Nashik	4,993	172	100%	First and solo		
Parbhani	1,527	202	100%	First and solo		
Pune	9,924	3,200	100%	Not first but solo	253	279
Sangli	2,583	400	54%	Not first but solo		
Satara	2,796	185	37%	First and solo		
Solapur	3,849	1,001	50%	Not first and only clinical services		
Yavatmal	2,460	204	100%	First and solo		

**Sources:** 1) Census 2001; 2) District mapping data; 3) Avahan’s computerized monitoring and information system (CMIS) 2009.

*First and solo: 1) Prior to Avahan (i.e., 2003), when there was no intervention for MSM/TG; **2)** Avahan was the only intervention in the district.

**Not first but major:** Prior to Avahan (i.e., 2003), when MSM interventions existed; currently Avahan covers more than 50% of the mapped HR-MSM/TG population in the district.

**Not first but equal:** Prior to Avahan (i.e., 2003), when MSM interventions existed; currently Avahan covers 30–50% of the mapped MSM population in the district.

**Not first and only clinical services:** Prior to Avahan (i.e., 2003), when MSM interventions existed; Avahan provides only clinical services in the district.

+Mumbai and Thane were together considered one domain for sampling.

^a^IBBA: Integrated behavioral and biological assessment.

## Methods

The Avahan program evaluation framework was adapted and used for this assessment (Table 2) [21]. We evaluated the impact of the program using this framework, based on the program logic model, and using data from two rounds of independent surveys and program monitoring data. The framework assesses the program in the following sequence: (1) program implementation fidelity to design (process and output indicators); (2) intermediate outcomes such as condom use and STIs in target populations, HIV prevalence in target population and general population; (3) role of high-risk interventions in any changes; and (4) Avahan’s contribution to these changes.

**Table 2:** **Avahan evaluation framework and the data sources used for evaluation**

Evaluation question	Indicator	Data source
**1. Is the coverage of Avahan adequate?**	**A. Scale**	
	a. ***Geographical coverage*** **:** Description of rollout in number of districts and change in the number of implementing NGOs over time	CMIS*
	b. ***Proportion of HR*** **-** ***MSM*** **/** ***TG ever contacted and ever visited clinic*** **:** Number of MSM ever contacted by Avahan peer educators or ever visited Avahan program STI clinics, divided by the estimated size of MSM as of March 2009	CMIS
	c. ***Proportion of MSM*** **c** ***ontacted monthly by peer educators or visited program STI clinics for STI consultations*** **:** Number of MSM contacted monthly by peer educators or visited program STI clinics monthly, divided by the estimated size of MSM as of March 2009	CMIS
	d. ***Proportion of MSM*** **/** ***TG contacted in the last month*** **:** Percentage of MSM from IBBA who reported that they had been contacted by Avahan peer educators in the month preceding the survey	IBBA**
	**B. Intensity**	
	a. ***Number of peer educator*** **/** ***outreach workers and ratio of MSM to peer educators*** **:** The total number of active outreach workers and peer educators in the Avahan intervention areas across implementation districts in Maharashtra; number of estimated MSM/TG covered per peer educator in the coverage area	CMIS
	b. ***Condom distribution and availability***	CMIS and condom social marketing data
	1. Absolute number of free condoms distributed by the Avahan program annually and condom sales from project-supported condom social marketing during 2005–2008	
	2. *Condom needs analysis*: Ratio of average monthly condoms available per MSM — total condoms distributed by Avahan and available through project-supported condom social marketing sales, divided by the estimated number of MSM in area covered by Avahan; ratio of number of condoms distributed to monthly commercial sex acts per MSM/TG, where sex acts are calculated based on the number of sex acts with paying and paid male partners per month, multiplied by the total estimated number of MSM covered by Avahan, multiplied by four to get monthly sex acts***.	
	3. Proportion of MSM reporting source of obtaining condoms last time from outreach worker/peer educator/NGO	IBBA
	c. ***Frequency of contact by peers*** **:** MSM/TG reporting the number of times they were contacted by peer educators in the month preceding the survey	IBBA
	d. ***Frequency of visit to clinic*** **:** MSM reporting the number of times they visited Avahan program clinics for STI services	Individual level CMIS data
**2. Has there been an increase in condom use in MSM?**	**Change in condom use pattern**	
	a. Proportion of MSM reporting last time condom use with paying male partners over the two rounds of IBBA	IBBA
	b. Proportion of MSM reporting consistent condom use with paid male partners over the two rounds of IBBA	IBBA
	c. Proportion of MSM reporting consistent condom use with regular male partners over the two rounds of IBBA	IBBA
	d. Proportion of MSM reporting consistent condom use with other non-commercial male partners over the two rounds of IBBA	IBBA
**3. Has there been a reduction in STI prevalence and new HIV infections?**	**Change in STI prevalence and visits to clinic with STI symptoms**	
	a. STI prevalence (reactive syphilis serology, high-titre syphilis, gonorrhoea (NG), chlamydia (CT), any STIs (NG, or CT or high-titre syphilis)	IBBA
	**Change in HIV prevalence and new HIV infections**	IBBA
	a. HIV prevalence among MSM aggregated from all districts in the two rounds of IBBA	
**4. Is Avahan exposure associated with increase in condom use and declining STIs?**	**Association of program exposure with intermediate outcomes and STI prevalence**	IBBA
	a. Program exposure is defined as exposure to any one of the following: ever contacted by Avahan peer educators, ever visited Avahan program clinic, and ever received condoms from peer educators; its association with consistent condom use with commercial and non-commercial partners using pooled data from the two rounds of IBBA	
	b. Duration of program exposure and its association with condom use with commercial and non-commercial partners using pooled data from the two rounds of IBBA	
	c. Program exposure, as defined above, and its association with presence of any STI (gonorrhoea, chlamydia or high-titre syphilis [*>1:8*])	

***In both rounds, MSM were asked about the number of times they had had anal sex with a paying male partner in the past one week; for paid male partners, it was the number of times they had anal sex in the past one month.

*CMIS: Avahan’s computerized management information system.

**IBBA: Integrated behavioral and biological assessment.

### A. Data sources

Data from three sources were used in this analysis to build evidence to assess the effect of Avahan on key programmatic outcomes: Avahan’s computerized management information system (CMIS), two rounds of cross-section surveys among HR-MSM/TG, called the Integrated Behavioral and Biological Assessment (IBBA); and program-supported condom social marketing data [22–24].

#### CMIS

Avahan’s CMIS [24, 25] provided data on program inputs, infrastructure, outreach, clinical service utilization, and community mobilization. This data were generated at the implementing organization level (local NGOs) and submitted to Avahan’s central CMIS by state-level lead implementing partners (LIPs). It was consolidated at district, LIP, state, and pan-Avahan levels. The consolidated monthly data from January 2005 through March 2009 for the 15 districts of Maharashtra was used to assess trends in coverage and uptake of program services. The coverage indicators included whether a high-risk group member was ever contacted by peer educators and had ever visited a program STI clinic. Avahan’s target for saturated coverage (defined as providing services to at least 80% of the estimated total population) in selected districts/catchment areas, assessed through participatory mapping and site assessment, was the benchmark against which the adequacy of coverage was assessed. The estimated size of the HR-MSM/TG population as of March 2009 (10,240) was used as the denominator for analysis. The service indicators examined were monthly peer contacts and quarterly clinic visits against Avahan’s established targets [outreach: one contact per month; clinic visits: once per quarter). Measures of program intensity included ratio of program staff (peers and outreach workers) to target population (against a target of 1:50 HR-MSM/TG) [see Table 2 for additional indicator details]. Detailed description of CMIS indicators and estimation process is available elsewhere [24].

#### IBBA

The IBBA surveys were conducted to measure the major outcomes and impact of HIV interventions supported by Avahan, the India initiative. In IBBA, HR-MSM/TG were operationally defined as men identified at cruising points, aged 18 years and older, who had had any type of sex (oral, anal, manual) with another man in the past month. Two rounds of cross-sectional IBBA surveys were conducted among HR-MSM/TG in three districts of Maharashtra — Mumbai, Thane and Pune, chosen purposively based on the overall study design of IBBA. The respondents were selected using the time location cluster sampling method [23]. Mumbai and Thane are neighboring districts, but for the purposes of the survey they were considered one domain due to high mobility of individuals between the two districts. Field work was conducted during January–April 2007 (Round 1) and in December 2009 (Round 2). Information on demographics, sexual behaviors, condom use, and exposure to intervention was collected in these surveys through a questionnaire. The participants were also tested for presence of STIs, including HIV. Written informed consent was obtained from respondents before administration of questionnaire and collection of sample for biological tests. The field work was conducted by research agencies under the guidance and supervision of National AIDS Research Institute (NARI), and FHI 360 (trade name for Family Health International) provided the technical support. Further details of the methodology are available elsewhere [23, 26–28] and additional IBBA-related information can also be accessed at http://www.ibbainfo.in. Data collection for IBBA was approved by the Protection of Human Subjects Committee (of FHI 360) and the ethical committee of NARI, an Indian Council of Medical Research (ICMR) institute.

#### Condom social marketing data

Annual condom sales data from Avahan-supported condom social marketing program [22] in Maharashtra (2005–2008) was used to assess condom distribution by the project and gaps in condom availability.

### B. Exposure and outcome variables

Four process and output indicators were assessed to inform the Avahan evaluation framework described above. These indicators were: (1) scale-up and intensity (programs reach and coverage) of Avahan coverage among HR-MSM/TG in Maharashtra; (2) reported consistent condom use; (3) prevalence of STIs, including HIV; and (4) associations between intermediate outcomes (condom use, prevalence of HIV/STIs) and exposure to the Avahan program (peer contacts, visits to NGO clinics and condom provision) [see Table 2 for indicator details].

#### Condom use

Self-reported condom use from the two rounds of IBBA was analyzed to assess changes in condom use patterns. Consistent condom use was defined as condom use during every act of anal intercourse with different types of male partners (such as, main regular male partners [lover/boyfriend/spouse], paying male partners [receiving cash or gifts when selling sex], paid male partners [giving cash or gifts to male partner or hijra when buying sex], and other casual male partners).

#### Sexually transmitted infections and HIV

Blood and urine samples were tested for STIs. Syphilis serology was done using rapid plasma reagin (RPR; by Span Diagnostics Ltd.) and confirmed by *Treponema pallidum* hemagglutination assay (TPHA; by Syphagen). Nucleic acid amplification (Gen-Probe APTIMA COMBO 2 – Gen-Probe Inc. San Diego, California) tests on urine samples were done for chlamydia and gonorrhoea. HIV positivity was determined by a serial testing algorithm using two rapid tests (Microlisa-IV by J. Mitra & Co. Pvt. Ltd. and Genedia HIV ½ ELISA 3.0 by Greencross Life Sciences) [23].

#### Program exposure

Avahan exposed the HR-MSM/TG to a combination of prevention interventions. Therefore, a composite indicator of selected intervention exposure — ever been exposed to any three core program services: contact by Avahan peer educators, visit to a Avahan program clinic, and provision of condoms from peer educator — was created and used for this analysis. We estimated the association between this composite indicator and consistent condom use with different male partners and STIs (including HIV).

### C. Data management and statistical analysis

Data from the two districts was combined and appropriate weights calculated and used for IBBA data analyses. All the analyses were performed in SPSS Version 15.01 (®IBM, New York). The proportions for categorical variables, means and 95% confidence intervals (CIs) for continuous variables were calculated. The difference between the proportions was tested using the Pearson chi-square test.

Logistic regression models were used to measure the association between exposure and outcome variables. We initially estimated the unadjusted associations between exposure and outcomes. Following this, we built multivariate models and adjusted for background characteristics and contextual variables related to geographical coverage that could confound results, such as district of interview, self-reported sexual identity, current age, literacy, place of residence, sex outside place of residence, and main source of income.

1. The first set of logistic regression models estimated the association between consistent condom use and STIs (including HIV) as outcomes and the round of IBBA data collection as the primary explanatory variables.
2. The next set of logistic models estimated the association between consistent condom use and STIs as outcomes and composite exposure to core program services of the Avahan intervention as explanatory variable.
3. For the third set of logistic regression models, we pooled data from both rounds of IBBA and evaluated the association between exposure to the intervention and condom use. Thereafter, these were analysed separately for rounds 1 and 2 of IBBA for consistent condom use (as an outcome) with different types of male partners. We compared the odds ratios in these two rounds of IBBA using the methods described by Altman and Bland [29].

## Results

A total of 1,305 HR-MSM/TG (653 in Round 1 and 652 in Round 2) were sampled. The mean age of HR-MSM/TG in Round 1 and Round 2 was 25.7 years (95% CI: 24.7 – 26.7) and 24.2 years (95% CI: 23.7 – 24.8), respectively (p = 0.001). HR-MSM/TG were more likely to be literate (96.4% vs. 89.4%, p = 0.01) and live locally (83.2% vs. 65.7%, p = 0.02) in Round 2 than in Round 1. Though HR-MSM/TG were less likely to report having had a paid male partner in Round 2 compared with Round 1 (8.6% vs. 29.9%, p = 0.001), they were more likely to have a non-commercial male/male-to-female transgender partner (71.9% vs. 51.3%, p = 0.001). HR-MSM/TG were less likely to report sex outside the place of current residence in Round 2 compared with Round 1 (33.3%vs. 23.2%, p = 0.04). HR-MSM/TG were more likely to identify themselves as bisexual (33.6% vs. 6.6%) and significantly fewer as *panthi* (locally used term for a male partner who mainly penetrates) in Round 2 compared with Round 1 (15.4% vs. 43.3%, p < 0.001). Select demographic characteristics, sexual behaviors, condom use, and exposure to the Avahan intervention in both rounds of IBBA are presented in Table 3.

**Table 3:** **Descriptive statistics (demographics, sexual behaviors, condom use, exposure to the program, and STIs, including HIV) of 653 and 652 HR-MSM/TG, respectively, in rounds 1 and 2 of IBBA, Maharashtra, India** ^**a**^

Variables		Round 1 (N = 653)%	Round 2 (N = 652)%	p value
**A. Socio-demographic profile**				
***Age*** **(** ***years*** **)**	18–24	62.0	46.4	0.02
	25–29	22.6	35.3	
	30–34	11.6	10.5	
	35–39	2.1	4.9	
	≥ 40	1.8	2.8	
***Literacy status***	Literate (can read and write)	89.4	96.4	0.01
	Illiterate	10.6	3.6	
***Marital status***	Ever married	18.1	23.1	0.26
	Never married	81.9	76.9	
***Main occupation***	Unemployed/student	17.9	17.4	0.09
	Self-employed/business/trade	22.6	29.0	
	Non-agricultural/agricultural labor	1.7	2.4	
	Service (government/private)	43.5	45.3	
	Sex work	11.3	3.8	
	Others (massagers/transport workers)	3.0	2.2	
***Lives locally***	Yes	65.7	83.2	0.02
	No	34.3	16.8	
**B. Sexual behaviors**				
***Age at first sexual exposure***	< 15 years	32.0	33.3	0.83
	≥ 15 years	68.0	66.7	
***Had sex outside the current area of residence***	Yes	23.2	33.3	0.04
	No	76.8	66.7	
***Types of sexual partners*** **(** ***ever*** **)**	Had regular male partner(s)	53.6	59.4	0.35
	Had paying male partner(s)	43.2	43.0	0.97
	Had paid male partner(s)	29.9	8.6	< 0.001
	Had other non-commercial male/transgender partner(s)	51.3	71.9	< 0.001
	Had regular female partner(s)	32.2	37.3	0.36
***Self*** **-** ***reported sexual identity*** ^***a***^	*Kothi*	27.5	39.0	< 0.001
	*Panthi*	43.3	15.4	
	*AC*/DC or double decker	16.0	9.7	
	*Bisexual*	6.6	33.6	
	*Hijra*	6.6	2.3	
**C. Condom use**				
***Condom use during the last sex act***	With regular male partner	65.9	91.3	< 0.001
	With paying male partner	81.0	98.3	< 0.001
	With paid male/hijra partner	84.8	57.7	0.01
	With other non-commercial male/hijra partner	82.2	80.3	0.75
***Consistent condom use***	With regular male partner(s)	51.6	62.0	0.23
	With paid male/hijra partner(s)	75.6	44.4	0.06
	With other non-commercial male/hijra partner(s)	60.2	68.7	0.37
**D. Exposure to the Avahan program interventions**				
	Contacted by Avahan peer educator/outreach worker	52.6	50.3	0.72
	Visited Avahan-supported clinic	28.3	46.3	0.005
	Received condoms from peer educator/outreach worker	56.7	49.9	0.28
	Exposed to Avahan intervention^b^	60.3	57.7	0.71
**E. Sexually transmitted infections (including HIV)**				
***Type of infections***	Reactive Syphilis Serology - Yes^†^	8.8	1.1	< 0.001
	Syphilis titre ≥ 1:8^††^	3.8	0.2	< 0.001
	Chlamydia infection – Yes	3.7	1.1	0.20
	*Neisseria gonorrhoea* infection - Yes	0.3	0.0	0.10
	HIV-1 infection – Yes	12.3	6.3	0.16

^a^ = All the proportions shown are weighted.

^†^ = Any person reactive on RPR and TPHA.

^††^ = The titre obtained on RPR was used to measure this variable.

^a^ *Kothis* are self-identified MSM who are generally feminine and receptive partners during anal sex; *panthis* are those who are masculine and insertive partners. The term ‘double-decker’ (also called ‘AC/DC’ in some study sites) refers to those men who are both insertive and receptive partners in anal sex. Even though *hijras* belong to a distinct socio-cultural group and can actually be regarded as male-to-female transgender people, they are included under the ‘MSM’ umbrella in this study.

^b^Defined as exposure to any one of the following: ever contacted by Avahan peer educator, ever visited Avahan program clinic, and ever received condoms from peer educators.

### Scale-up of Avahan program

**Figure 1:**
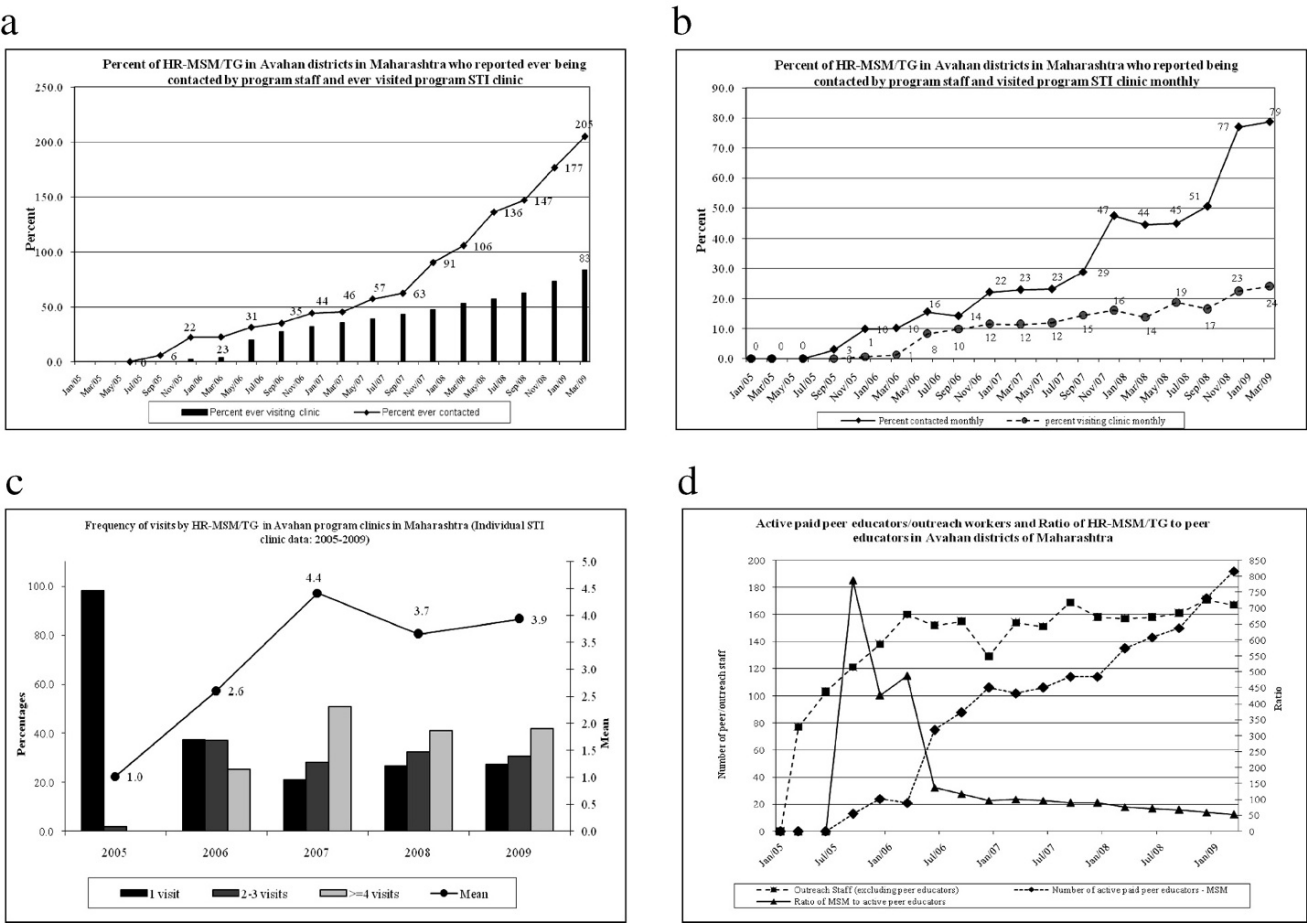
**Scale and coverage of different components of the Avahan programme in high-risk men who have sex with men and transgendered persons (HR-MSM/TG) Maharashtra, India 2005-2009. a)** Exposure to Avahan program; **b)** Monthly contact by program staff and visit to the STI clinic; **c)** Frequency of visits to Avahan STI clinic; **d)** Peer educators / Outreach workers from Avahan districts.

The Avahan program for HR-MSM/TG in Maharashtra began in late 2004 in three districts and scaled up to 15 districts by 2008 through 20 local implementing NGOs working under state LIPs. The CMIS data in December 2005 indicated that 22% of the estimated HR-MSM/TG had ever been contacted; however, scale-up increased this coverage to 90% by December 2007 and continued to increase consistently until March 2009. Those HR-MSM/TG who had ever visited STI clinics increased from 20% in mid-2006 to 83% in March 2009 (Figure 1a). The proportion of HR-MSM/TG contacted monthly by peer educators increased from 10% in early 2006 to about 79% in March 2009, and monthly STI clinic visits increased from 1% to 24% during the same period (Figure 1b). There was a considerable increase in the mean frequency of visits by HR-MSM/TG to STI clinics from 2005 (mean: 1.0) to 2009 (mean 3.9). About 42% of HR-MSM/TG reported four or more clinic visits in 2009 (Figure 1c).

The number of active paid peer educators was low in the initial stages of the program (13 peer educators in mid-2005) but increased as the program was scaled up (192 by March 2009). Similarly, the number of outreach workers increased from 77 in early 2005 to 167 in March 2009 (Figure 1d). The ratio of HR-MSM/TG to peer educators was not optimal during the initial years but stabilized to a level of 1:53 by March 2009 (Figure 1d).

Free direct condom distribution by Avahan program NGOs in Maharashtra increased from about 0.37 million condoms in 2005 to 1.8 million in 2008. During the same period, condoms distributed by Avahan-supported social marketing outlets in program districts increased from more than 2.6 million in 2004 to about 14.4 million in 2008. The monthly ratio of free condoms distributed directly to HR-MSM/TG covered by the program increased from two condoms per HR-MSM/TG per month in 2005 to 15 per HR-MSM/TG per month in 2008.

### Condom use

The HR-MSM/TG were significantly more likely to report last time (last instance of) condom use (adjusted odds ratio [AOR]: 5.15; confidence interval [CI] 2.68-9.90) as well as consistent condom use (AOR: 1.90; CI 1.01-3.58) with regular male partners in Round 2 of the IBBA than in Round 1. Similarly, HR-MSM/TG were more likely to report condom use during last sex act with a paying partner in Round 2 of IBBA than in Round 1 (AOR: 13.23; CI 2.84-61.92). Although in univariate analysis HR-MSM/TG were less likely to use condoms during last sex act with a paid male/hijra partner in Round 2 compared with Round 1, this difference was not significant (AOR: 0.91; CI 0.27-3.10) after adjustment for potential confounders. Consistent condom use with a regular female partner was low in both rounds of IBBA (33.6% in Round 1 and 25.2% in Round 2, p = 0.39). Information on condom use with different types of male partners is presented in Tables 3 and 4.

**Table 4:** **Association between outcomes (condom use and STIs, including HIV) and rounds of IBBA, Maharashtra, India** ^**a, b**^

Variables		Odds ratio (95% CI)	Odds ratio (95% CI)
		Unadjusted models	Adjusted models^c^
**A. Condom use**			
***Condom use during the last sex act***	With regular male partner	1.93 (1.48 – 2.53)**	5.15 (2.68 – 9.90)**
	With paying male partner	13.77 (2.94 – 64.59)**	13.23 (2.84 – 61.92)**
	With paid male/hijra partner	0.24 (0.08 – 0.73)*	0.91 (0.27 – 3.10)
	With other non-commercial male/hijra partner	0.88 (3.16 – 6.74)	0.71 (0.27 – 1.85)
***Consistent condom use***	With regular male partner(s)	1.07 (0.83 – 1.38)	1.90 (1.01 – 3.58)*
	With paid male/hijra partner(s)	0.26 (0.06 – 1.10)	0.57 (0.16 – 2.08)
	With other non-commercial male/hijra partner(s)	1.45 (0.64 – 3.31)	1.71 (0.82 – 3.58)
**B. Sexually transmitted infections (including HIV)**			
***Type of infections***	Reactive Syphilis Serology†	0.12 (0.05 – 0.25)**	0.11 (0.05 – 0.22)**
	Syphilis titre > 1:8 ††	0.06 (0.02 – 0.19)**	0.08 (0.03 – 0.27)**
	Chlamydia infection	0.04 (0.02 – 0.07)**	0.35 (0.05 – 2.41)
	*Neisseria gonorrhoea* infection	0.003 (0.001 – 0.010)	--
	HIV 1 infection	0.48 (0.17 – 1.34)	0.39 (0.17 – 0.87)*

^a^ = The estimates shown here are weighted estimates.

^b^ = The reference for each of the estimate is Round 1 of the IBBA. Thus, for consistent condom use with a regular male partner, the interpretation will be as follows: subjects in Round 2 of IBBA were significantly more likely to report consistent condom use with a regular male partner, compared with those in Round 1 of IBBA (OR: 1.90, 95% CI: 1.01 – 3.58).

^c^ = The models were adjusted for district of interview, self-reported sexual identity, current age, literacy, place of residence, sex outside place of residence, and main source of income.

*p = 0.05, ** p < 0.01.

^†^ = Any person reactive on RPR and TPHA.

^††^ = The titre obtained on RPR was used to measure this variable.

Furthermore, HR-MSM/TG who were exposed to various components of the Avahan intervention were significantly more likely to report consistent condom use with regular male partners (AOR: 2.46; CI 1.34-4.52), non-commercial male/hijra partners (AOR: 3.41; CI 1.56-7.50), and paid male/hijra partners (AOR: 3.15; CI 1.37-7.25), compared to those who had not been exposed to the intervention (Table 5).

**Table 5:** **Association between condom use (during last sex act and consistent condom use) and exposure to various components of the Avahan program intervention in rounds 1 and 2 of IBBA, according to different types of male partners, Maharashtra, India** ^**a, b**^

Variables		Percentages		Odds ratios	
		Exposed to Avahan intervention	Not exposed to Avahan intervention	Unadjusted models odds ratio (95% CI)	Adjusted models^c^odds ratio (95% CI)
**Condom use during the last sex act**	With regular male partner	80.9	76.9	1.27 (0.68 – 2.38)	1.17 (0.64 – 2.14)
	With paying male partner	89.3	88.0	1.13 (0.53 – 2.44)	0.75 (0.39 – 1.43)
	With paid male/hijra partner	83.8	75.5	1.68 (0.58 – 4.86)	2.34 (0.60 – 9.11)
	With other non-commercial male/hijra partner	79.5	84.0	0.74 (0.35 – 1.57)	0.73 (0.36 – 1.50)
**Consistent condom use**	With regular male partner(s)	74.2	57.5	2.68 (1.36 – 5.29)**	2.46 (1.34 – 4.52)**
	With paid male/hijra partner(s)	76.5	63.4	1.89 (0.81 – 4.38)	3.15 (1.37 – 7.25)**
	With other non-commercial male/hijra partner(s)	73.9	50.1	2.82 (1.25 – 6.36)*	3.41 (1.56 – 7.50)**

^a^ = The estimates shown here are weighted estimates.

^b^ = The reference for each of the estimate is not being exposed to that particular component of the program. Thus, for consistent condom use with a regular male partner, the interpretation will be as follows: subjects who were exposed to Avahan program were more likely to report consistent condom use with a regular male partner compared with those who were not contacted by the peer (adjusted OR: 2.46, 95% CI: 1.34 – 4.52).

^c^ = The models were adjusted for district of interview, self-reported sexual identity, current age, literacy, place of residence, sex outside place of residence, and main source of income.

* = p < 0.05, ** = p < 0.01.

### STIs

Overall, prevalence of reactive syphilis serology declined from 8.8% in Round 1 of IBBA to 1.1% in Round 2 (p = 0.001); a similar decline was also observed in high-titre syphilis (3.8% in Round 1 vs. 0.2% in Round 2). Prevalence of urethral chlamydial (3.7% in Round 1 vs. 1.1% in Round 2) and gonococcal infection (0.3% in Round 1 vs. 0.0% in Round 2), was low in Round 1 and reduced further in Round 2; no cases of urethral gonorrhoea were reported in Round 2. The observed change in HIV prevalence was not statistically significant (12.3% in Round 1 vs. 6.3% in Round 2, p = 0.16).

In terms of district-wise data, prevalence of syphilis declined in Pune from 14.6% to 5.0% (p = 0.001) as did HIV prevalence, from 17.4% in Round 1 to 8.2% in Round 2 (p = 0.01). Similarly, in the combined domain of Mumbai and Thane, prevalence of syphilis reduced from 6.5% in Round 1 to 0.5% in Round 2 (p = 0.001). However, the observed change in HIV prevalence in Mumbai-Thane was not statistically significant (10.2% in Round 1 vs. 6.0% in Round 2, p = 0.36).

## Discussion

This paper is the first to present findings from a large-scale assessment of HR-MSM/TG in Maharashtra, following Avahan’s HIV-prevention interventions. The Avahan intervention was able to achieve the targeted 80% coverage of the HR-MSM/TG population in Maharashtra. The effect of this exposure to prevention interventions was evident in the target group. A significantly higher proportion of HR-MSM/TG reported attending the STI clinic in 2009, compared to 2006. Self-reported consistent condom use with regular male partners was significantly higher in 2009 than in 2006. HR-MSM/TG who were exposed to the Avahan intervention were more likely to report consistent condom use with various types of male partners than those who were not exposed to the intervention. Prevalence of reactive syphilis serology reduced significantly in 2009 compared with 2006; however HIV prevalence did not change over time.

The findings suggest that Avahan was successful in accessing the HR-MSM/TG population in selected districts, increasing the number of outreach workers and peer-led contacts with HR-MSM/TG, improving outreach, and increasing the distribution and access of condoms for this group. It is important to mention here that not only were these targets achieved but they were also sustained for the duration. A higher than 100 percent coverage, as reflected in the CMIS data, may likely be due to the highly mobile nature of the population; the denominator was fixed to the estimated population in March 2009 whereas the numerator (members of population) was cumulative, reflecting individual HR-MSM/TG contacted over the course of the intervention (this included members who are currently present or who may have been present at an earlier time but subsequently left the area). Similar high coverage was observed among FSWs in Avahan implementation districts of Maharashtra [30]. Indeed, in Andhra Pradesh a similar successful scale-up of the HR-MSM/TG intervention has also been reported [31]. In both these scenarios, the target reached was much higher than the global average of 31% outreach documented for low and middle-income countries [32]. However, we would like to state here that Avahan attempted to cover the visible MSM, who are presumably at a high risk and reachable; the percent coverage may thus be higher than the global percentage. Nonetheless, the high coverage in this group is important given the fact that targeted interventions had started as late as 1999–2000 among the MSM population in Maharashtra.

In addition to the improved infrastructure for HIV prevention for HR-MSM/TG, changes in risk behaviors are also evident. Study findings reveal that HR-MSM/TG reported higher condom use during the last sex act with a regular and paying male partner in the second round of IBBA. Furthermore, HR-MSM/TG who were exposed to the Avahan intervention were more likely to report consistent condom use with a regular male partner. The increased availability of condoms during the same period supports this finding. Although condom use during sex may be dependent on factors other than accessibility (such as negotiation and breakage), access to condoms does form an important component of HIV interventions [33]. However, decline in both condom use with paid partners during last sex and consistent condom use was observed; similarly, consistent condom use with female partners was low in both rounds of IBBA. An important risk factor associated with unprotected sex was mobility of the individual; MSM who were mobile were more likely to report unprotected sex with male partners compared with non-mobile MSM [34]. Thus, further analysis and studies are needed to better understand the factors affecting HR MSM’ and TGs’ condom use patterns with different partners, which would help HIV prevention programs develop more effective behavior change communication strategies. Low condom use with female partners, reported by other studies as well [4, 11], is an important area of concern for HIV prevention programs working with MSM/TG. Given that a sizable proportion of the MSM population has female partners, low condom use has significant implications for HIV transmission to general population [11]; HIV prevention programs must therefore take this into consideration when planning targeted interventions. Decline in condom use with paid partners could have been due to non-usage of condoms by *kothis* (locally used term for self-identified MSM who are generally feminine and receptive partners during anal sex) and bisexuals, who were the majority in Round 2. Also, bisexuals are usually difficult to identify and not covered by prevention programs. Thus, future programs targeting risk reduction should be more tailored to the local dynamics of MSM/TG, cover all MSM irrespective of their sexual identities, and be sensitive and culturally acceptable.

Another important finding was the reduction in syphilis prevalence over the three years’ duration. Focussed STI-prevention measures and proper management of STIs through dedicated clinics may have led to this decline in prevalence. Improved access to STI clinics is important for HIV prevention since prompt treatment of ulcerative and non-ulcerative STIs are useful in reducing the transmission of HIV in this high-risk group [35, 36]. Furthermore, STI clinics serve as an entry point for counselling, behavioral change, and health care access in general. A recent study also reported that a structured intervention (which included risk reduction counselling and self-acceptance among MSM) has also been useful in reduction of incident bacterial STIs [37]. Thus, this entry point becomes particularly relevant for marginalized populations like MSM/TG, who may have poor health care access due to stigma and discrimination [33, 38].

While, the HIV prevalence remained unchanged, the estimated prevalence among HR-MSM/TG reported here is lower than that reported by earlier studies [10, 15, 39] and the data is comparable to national HIV surveillance (2008–09) [40]. In one of the earliest reports (mid-1990s), Nandi and co-workers found HIV prevalence to be 21% in a sample of MSM in Mumbai [39]. Several factors influence HIV prevalence and it is a short period of time between the two IBBA surveys to plausibly associate any change in HIV following programmatic interventions.

Nonetheless, the present study does have a number of limitations. The first round of IBBA was conducted one year after the initiation of the Avahan intervention and therefore not a true baseline. Avahan’s evaluation design did not include a control group, given the ethical considerations to be applied for large-scale public health programs. However, the used evaluation framework did follow program logic, and data from multiple sources (both program monitoring and independent survey data) was used to suggest plausible evidences for the effectiveness of the intervention, as alternative to randomised controlled trials, which are now considered less suitable for large public health interventions [41–44]. Although Avahan was a pan-Maharashtra intervention, the IBBA surveys were restricted to three districts; while the CMIS data shows the scale up and coverage of the program across all districts, the IBBS data provides outcomes in a sample of districts, providing confidence on validity of the monitoring data. Another limitation in IBBA was that gonorrhoea and chlamydia were tested based on urethral samples, as rectal samples could not be collected in the given field conditions. Other studies from India, where throat or rectal swabs were collected, prevalence of oropharyngeal and rectal gonorrhoea was 4.7% and 7.4%, respectively [45]. Other limitations include those applicable to cross-sectional studies. For example, there were changes in population profile over the two rounds of the survey; however, these were accounted for by controlling for changes in multivariate models. Social desirability bias for self-reported behaviors (such as condom use) is a limitation, but it was presumed that such bias was similar for both rounds of IBBA.

In spite of these limitations, this assessment has several strengths. It is one of the first assessments of a large public health intervention for HR-MSM/TG in Maharashtra. The analysis was based on Avahan’s evaluation framework and evidence presented along the program logic model, examining a sequence of infrastructure, intermediate outcomes (behavior change and STIs), and the role of the intervention in these changes, thereby providing congruency of expected trends.

## Conclusions

The Avahan program in Maharashtra for HR-MSM/TG was successfully scaled up to achieve its targets through outreach, condom distribution and STI clinical services. The current evaluation shows that Avahan program effectively covered the target group, improved accessibility to condoms and reduced risk behaviours with male sexual partners. Syphilis prevalence declined; however HIV prevalence did not change and therefore a major concern. However, the evaluation also suggests that additional tailored and innovative behaviour change interventions are required to increase condom use behaviours with all types of partners, so as to reduce risk among HR-MSM/TG. Continued strengthening of core programmatic strategies will help bring sustained reductions in HIV risk in HR-MSM/TG and check its onward transmission.

## Abbreviations

HIV: Human immunodeficiency virus
STI: Sexually transmitted infection
HR-MSM/TG: High-risk men who have sex with men and transgender
FSW: Female sex worker
IDU: Injecting drug user
NACO: National AIDS Control Organisation
CMIS: Computerized management information system
IBBA: Integrated behavioral and biological assessment
NGO: Non-governmental organization
LIP: Lead implementing partners
NARI: National AIDS Research Institute
PHSC: Protection of Human Subjects Committee
ICMR: Indian Council of Medical Research.
